# Vascular Injury Patterns in High-Energy Trauma: A Systematic Review of Incidence, Diagnostic Modalities, and Management Strategies

**DOI:** 10.7759/cureus.91761

**Published:** 2025-09-07

**Authors:** Muhammad Zain Ul Abidin, Murhaf Assaf, Ahmed A Ali, Shenouda Shehata Abdelmesih, Mohammed M Alsharif, Moazer Ibrahim Hamid Mohammed, Abdelrahman Ibrahim, Aliaa H Alkhazendar, Faiqa Ijaz, Manahil Awan, Ahmed Khan

**Affiliations:** 1 Trauma and Orthopaedics, Luton and Dunstable University Hospital, Luton, GBR; 2 General Surgery, Princess of Wales Hospital, Cwm Taf Morgannwg University Health Board, Bridgend, GBR; 3 Emergency, Mubarak Al-Kabeer Hospital, Jabriya, KWT; 4 Orthopaedics and Traumatology, Royal Gwent Hospital, Newport, GBR; 5 Orthopaedics and Traumatology, Khoula Hospital, Muscat, OMN; 6 General Surgery, Al-Ahli Hospital, Hebron, PSE; 7 Surgery, BUPA (British United Provident Association) Arabia, Jeddah, SAU; 8 Trauma and Orthopaedics, University Hospitals of North Midlands NHS Trust, Stoke-on-Trent, GBR; 9 Surgery, The Islamic University of Gaza, Gaza, PSE; 10 Medicine, Fatima Jinnah Medical University, Lahore, PAK; 11 Executive and Special Ward, Liaquat National Hospital, Karachi, PAK; 12 Medicine, Elizabethtown College, Islamabad, PAK

**Keywords:** ct angiography, endovascular repair, high energy trauma, incidence, limb salvage, vascular injury

## Abstract

Vascular injuries in high-energy trauma are life- and limb-threatening emergencies, requiring prompt diagnosis and intervention. This systematic review included five studies published between 2019 and 2023, comprising approximately 480 patients. A literature search of PubMed/MEDLINE, Embase, Scopus, and the Cochrane Library was conducted using keywords such as “vascular injury,” “high-energy trauma,” “CT angiography,” and “endovascular repair.” Most studies focused on extremity injuries, particularly involving the femoral, popliteal, iliac, and brachial arteries. CT angiography (CTA) consistently demonstrated superior diagnostic accuracy compared to Doppler ultrasonography or clinical examination. Endovascular techniques, such as stenting and embolization, were compared with open surgical repair. Outcomes assessed included amputation rates, limb salvage, mortality, and hospital stay. Endovascular methods showed potential benefits in reducing complications and hospital duration in selected patients. Common pathophysiological patterns included arterial disruption, thrombosis, and ischemia-reperfusion injury. However, none of the studies evaluated renal outcomes. The included studies were assessed for risk of bias using ROBINS-I (Risk of Bias in Non-randomized Studies of Interventions) and the Newcastle-Ottawa Scale (NOS), revealing moderate quality overall. These findings highlight the diagnostic value of CTA and suggest a growing role for endovascular management in high-energy vascular trauma. Further high-quality studies are needed to guide treatment selection and optimize outcomes.

## Introduction and background

High-energy trauma injuries represent a unique and often challenging population. High-energy trauma, such as that resulting from motor vehicle collisions, falls from height, and industrial accidents, is a significant cause of morbidity and mortality [1]. Vascular injuries of the upper extremity are relatively rare, representing only about 1% of all traumatic injuries. However, they account for 30% to 40% of arterial trauma cases occurring in the upper extremities. Additionally, major torso vascular injury is found in less than 1% of trauma cases. Despite being rare, major vascular injuries are life-threatening, often require emergent intervention, and are associated with a 10%-20% mortality rate [2].

Vascular trauma can occur in three forms: blunt, penetrating, or combined [3]. It may result in significant morbidity and mortality when associated with bone, nerve, or soft-tissue damage. Blunt vascular trauma frequently accompanies complex musculoskeletal injury, especially in younger adults, and is a strong predictor of limb loss and functional deficits [4].

The diagnosis of vascular injuries depends on a combination of clinical assessment and radiological imaging. There are two types of signs when considering vascular trauma: hard signs and soft signs. Hard signs, such as pulsatile bleeding, rapidly enlarging hematoma, absent distal pulses, a cold or pale extremity, palpable thrill, and audible bruit, indicate an urgent vascular injury. Soft signs, including peripheral nerve deficits, a history of moderate hemorrhage, diminished but palpable pulses, or wounds near major arteries, necessitate further evaluation with imaging modalities such as FAST (Focused Assessment with Sonography for Trauma), ultrasound, chest X-ray, or CT angiography (CTA). Among these, CTA has become the gold standard for polytrauma cases, offering a sensitivity of 95%-100% [5]. FAST is a non-invasive tool that combines B-mode and Doppler ultrasound for rapid assessment. Given its high sensitivity for detecting arterial injuries (95%-97%), it has largely replaced conventional angiography for initial screening and triage. However, when vascular lesions are confirmed, formal angiography is often performed prior to surgery to precisely characterize the lesion and map the vascular anatomy [6].

The mechanism of injury, whether blunt or penetrating, significantly impacts both the pattern of vascular damage and the chosen treatment strategy. Management of vascular injuries remains highly complex, with the primary goals being hemorrhage control and restoration of blood flow to prevent mortality and limb loss. Torso vascular injuries pose an immediate life-threatening risk, while peripheral injuries threaten limb viability. Treatment approaches have evolved from traditional open repair to more advanced endovascular, hybrid, and selective non-operative techniques. For thoracic aortic injuries, thoracic endovascular aortic repair (TEVAR) is now considered the preferred method [7]. Emerging evidence supports endovascular management, including stenting or balloon occlusion (e.g., resuscitative endovascular balloon occlusion of the aorta (REBOA)), as effective alternatives to open repair, potentially reducing surgical morbidity and hospitalization times. Nevertheless, comprehensive analyses comparing outcomes remain limited. This review synthesizes the current literature on vascular injury patterns in high-energy trauma, diagnostic accuracy, and therapeutic outcomes.

## Review

Materials and methods

Search

This systematic review adhered to the Preferred Reporting Items for Systematic Reviews and Meta-Analyses (PRISMA) 2020 guidelines to ensure methodological rigor [8]. A comprehensive search was performed across PubMed/MEDLINE, Embase, Scopus, and the Cochrane Library through June 2025. Search terms and MeSH included “vascular injury,” “high-energy trauma,” “CT angiography,” “endovascular repair,” “open vascular repair,” “limb salvage,” and “trauma.” Boolean operators were applied to refine sensitivity and specificity. Human, English-language studies were included. The search was limited to studies published in English up to June 2025.

Eligibility Criteria

The eligibility criteria were defined using the PICO framework [9]. Patients (P) included those with vascular injuries from high-energy trauma. Interventions (I) were diagnostic methods, mainly CTA, and treatments such as endovascular or open repair. Comparators (C) involved endovascular versus open surgical approaches, and outcomes (O) assessed were incidence, diagnostic accuracy, amputation rates, mortality, and hospital stay. Studies were included if they involved human subjects with confirmed vascular injury, used cohort or case-series designs, provided comparative data on repair methods, reported quantitative outcomes, and were available as full-text, English-language articles. Exclusions applied to case reports, editorials, conference abstracts, animal studies, studies without clear outcomes, and those focused on low-energy trauma or isolated musculoskeletal injuries.

Study Selection

Two reviewers independently evaluated the titles and abstracts to determine relevance. Full-text versions of studies deemed potentially eligible were subsequently reviewed for inclusion. Any disagreements were resolved through discussion or, when necessary, by involving a third reviewer. The study-selection process was documented using a PRISMA flowchart.

Data Extraction

Two reviewers independently extracted data, including study design, sample characteristics, injury mechanism, diagnostic tools, management approaches, outcomes (e.g., limb loss, mortality, hospital stay), and the duration of follow-up.

Risk of Bias Assessment

The assessment of bias risk was carried out using ROBINS-I (Risk of Bias in Non-randomized Studies of Interventions), specifically designed for non-randomized studies [10]; the AMSTAR-2 (A Measurement Tool to Assess Systematic Reviews, version 2) checklist for narrative reviews [11]; and the Newcastle-Ottawa Scale (NOS) for observational cohorts [12]. Each study was rated independently by two reviewers, with consensus resolution for discrepancies.

Data Synthesis

Due to variability in study designs, populations, and reported outcomes, a meta-analysis was not feasible; therefore, a narrative synthesis was performed. Findings were grouped by incidence, diagnostic methods, and management strategies. Where comparative data existed, outcomes of endovascular versus open repair - such as amputation rates, mortality, and hospital stay - were summarized qualitatively to highlight key trends and differences.

Results

Study Selection Process

**Figure 1:**
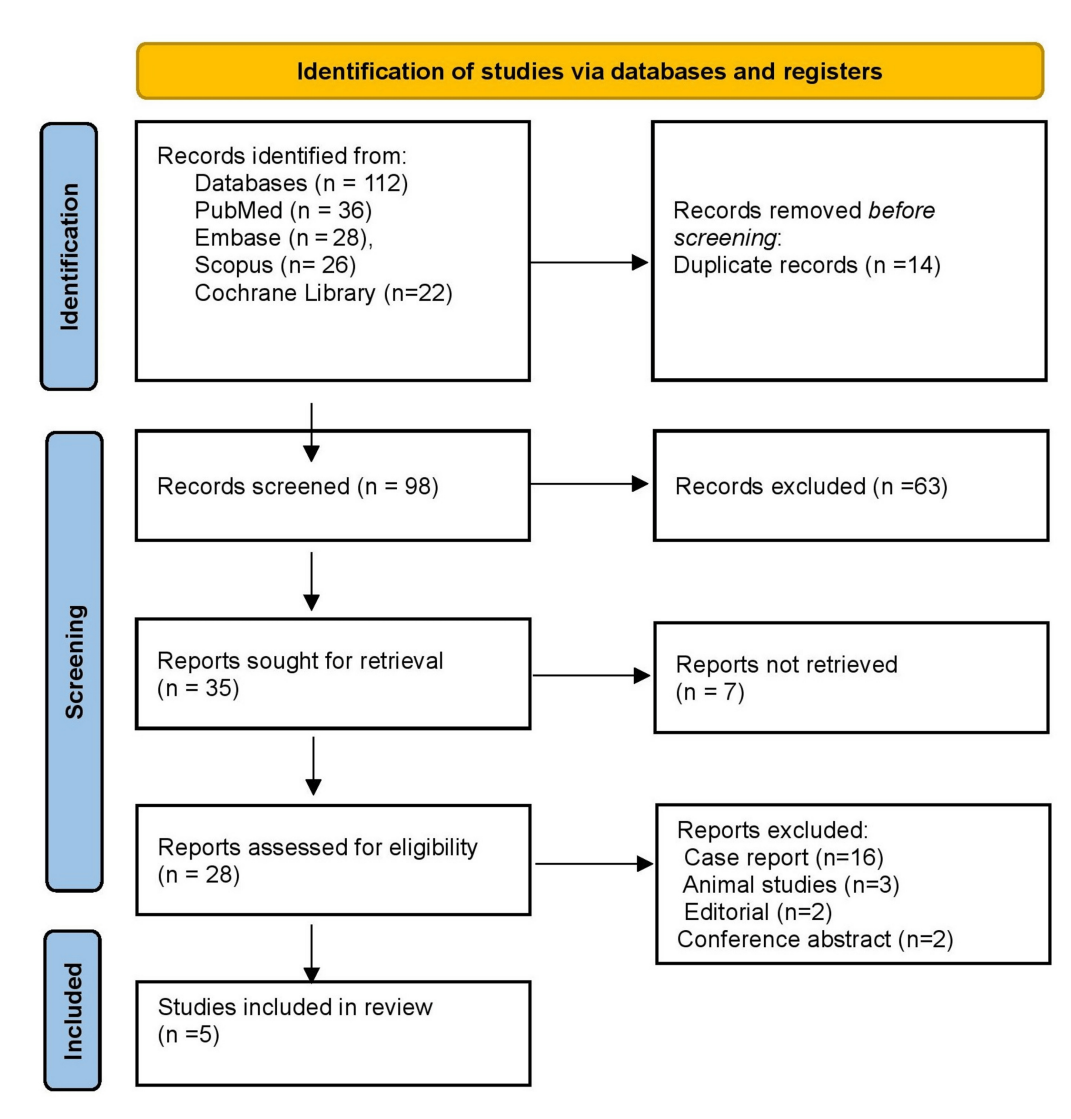
Study selection process through PRISMA guideline 2020 PRISMA, Preferred Reporting Items for Systematic Reviews and Meta-Analyses

Figure 1 shows that a total of 112 studies were retrieved from multiple databases: PubMed/MEDLINE contributed 36 records, Embase 28, Scopus 26, and the Cochrane Library 22. After removing 14 duplicates, 98 records were screened based on titles and abstracts, of which 63 were excluded due to irrelevance or insufficient data. The full texts of 35 articles were assessed for eligibility; however, seven reports could not be retrieved, despite repeated attempts. Of the 28 full-text studies reviewed, 23 were excluded for reasons such as being case reports, editorials, conference abstracts, or animal studies. Ultimately, five studies fulfilled all criteria and were included in the final review.

Characteristics of the Selected Studies

The characteristics of the selected studies are summarized in Table 1. The five studies, published between 2014 and 2021, collectively analyzed over 1,200 patients, with sample sizes ranging from 61 to more than 700. Most studies emphasized the diagnostic value of CTA, which proved highly effective for early detection of vascular injuries, compared to conventional diagnostic protocols. Several studies compared endovascular repair techniques, such as stenting and embolization, with open surgical repair, reporting outcomes including limb salvage, amputation rates, hospital stay, and procedural complications. Common pathophysiological findings included arterial disruption, thrombosis, pseudoaneurysm formation, and ischemia-reperfusion injury, with the femoral, popliteal, and brachial arteries frequently involved. None of the studies assessed renal outcomes, as the primary focus was on diagnostic accuracy and the effectiveness of repair strategies.

**Table 1:** Characteristics of the selected studies CT: Computed Tomography; ED: Emergency Department

Authors & Year	Population (P)	Exposure/Condition (I)	Comparator (C)	Outcomes (O)	Pathophysiological Findings	Anatomical Impact
Asmar et al. (2021) [13]	Trauma patients with peripheral arterial injuries (n ≈ 786)	Endovascular repair (stenting, embolization)	Open surgical repair	Amputation-free survival, complications, length of stay, 30-day readmission	Ischemia, repair site thrombosis, seroma	Axillary, brachial, femoral, popliteal arteries
Potter et al. (2021) [14]	Patients with superficial femoral/popliteal arterial trauma	Endovascular repair	Open surgical repair	In-hospital amputation, survival	Vessel injury with limb ischemia	Superficial femoral & popliteal arteries
Muckart et al. (2014) [15]	Blunt polytrauma patients (n = 1,033; 61 with vascular injury)	CT angiography for blunt vascular injury diagnosis	No comparator (descriptive study)	Incidence (5.9%), amputation rate (48%), mortality (28%)	Delayed ischemia, hemorrhage, delayed presentation	Extremity, thorax, abdomen, head/neck vasculature
Tamburrini et al. (2024) [16]	Review of ED trauma cohorts (diagnostic review)	CT angiography diagnostic protocols	Conventional or delayed imaging	Diagnostic sensitivity, detection rate, pitfalls	Intimal tears, pseudoaneurysm, vessel transection	Peripheral arteries, extremity vasculature
O'Banion and Magee (2022) [17]	Civilian vascular trauma registry (n ≈ several hundred)	Trends over time in treatment approach	Endovascular vs. open repair	Limb salvage, fasciotomy, complications	Variable ischemia and injury severity	Extremity and junctional arterial injuries

Risk of Bias Assessment

For the five included studies, the ROBINS-I tool was applied to the retrospective cohorts and registry-based analyses, Asmar et al. [13], Potter et al. [14], and Muckart et al. [15], while the NOS was used for the observational cohort by Muckart et al. [15]. The narrative review by Tamburrini et al. [16] was assessed using the AMSTAR-2 checklist. All studies were independently evaluated by two reviewers, with any discrepancies resolved through discussion and consensus (Table 2).

**Table 2:** Risk of bias assessment of the selected studies ROBINS-I: Risk of Bias in Non-randomized Studies of Interventions; NOS: Newcastle-Ottawa Scale; AMSTAR-2: A Measurement Tool to Assess Systematic Reviews, version 2

Study	Study Design	Risk of Bias Tool	Risk of Bias Rating	Justification
Asmar et al. (2021) [13]	Retrospective cohort	ROBINS-I	Moderate	Non-randomized design, potential confounders despite propensity matching
Potter et al. (2021) [14]	Retrospective cohort	ROBINS-I	Moderate	Similar dataset to Asmar; selection bias possible, though well-reported outcomes
Muckart et al. (2014) [15]	Observational cohort	Newcastle-Ottawa Scale (NOS)	Moderate	Clear inclusion criteria but limited adjustment for confounding variables
Tamburrini et al. (2024) [16]	Narrative review	AMSTAR-2	Low	Narrative synthesis only, lacking systematic review methodology
O'Banion and Magee (2022) [17]	Registry-based analysis	ROBINS-I	Moderate	Potential missing data and reporting bias due to the retrospective nature

Discussion

Patterns of vascular injury in high-energy trauma vary across the torso, neck, and peripheral regions, reflecting both the mechanism and anatomical exposure. Torso vascular injuries, such as thoracic aortic or iliac disruptions, are relatively rare but carry high mortality. Muckart et al. [15] observed that in blunt polytrauma, vascular injuries occurred in 5.9% of patients, with mortality rates of approximately 28%. TEVAR is the treatment of choice for blunt thoracic aortic injuries, as it significantly reduces perioperative mortality compared to traditional open surgical repair [18]. REBOA is also gaining traction for temporary hemorrhage control in severe torso injuries [19]. Neck vascular injuries, although less frequently reported in the reviewed studies, share similar diagnostic strategies. Tamburrini et al. [16] emphasized the diagnostic accuracy of CTA, which has effectively replaced conventional angiography for both blunt and penetrating neck vascular injuries, due to its near 100% sensitivity. Modern imaging protocols, including multidetector CTA, facilitate the early identification of intimal tears, pseudoaneurysms, and dissections. Endovascular stenting of carotid and vertebral arteries is increasingly employed in select cases, with meta-analyses reporting reduced stroke risks and avoidance of high-morbidity open procedures [20].

Peripheral vascular injuries are the most common, particularly in the extremities, due to their superficial location. Both Asmar et al. [13] and Potter et al. [14] demonstrated that femoral, popliteal, and brachial artery injuries are prevalent in high-energy mechanisms, and that endovascular repair offers favorable limb salvage rates, shorter hospital stays, and lower complication rates compared to open repair. O’Banion and Magee [17] further noted that treatment strategies should be tailored according to the vascular bed, with hybrid approaches combining open and endovascular techniques in complex injuries. The mechanism of trauma is a key determinant of injury patterns. Penetrating trauma often causes direct vessel laceration or transection, whereas blunt trauma leads to intimal flaps, thrombosis, or delayed pseudoaneurysm formation. Muckart et al. [15] highlighted delayed presentation of vascular injuries in blunt trauma cases, contributing to high amputation rates (48%). This underscores the importance of prompt vascular imaging, particularly CTA, as highlighted by Tamburrini et al. [16].

Overall, the evolving preference for endovascular or hybrid approaches represents a paradigm shift in the management of both central and peripheral vascular injuries. Large multicenter studies and registries, such as those cited by Asmar et al. [13], support the safety and efficacy of endovascular interventions for peripheral arterial injuries. However, open repair remains essential in cases of complete vessel transection, contamination, or severe musculoskeletal injury. Future studies should compare long-term patency and functional outcomes between open, hybrid, and endovascular techniques. This review is limited by the small number of included studies, heterogeneity in reporting outcomes, and the lack of randomized trials, which restricts the ability to generalize findings or perform a meta-analysis.

## Conclusions

High-energy trauma results in distinct vascular injury patterns across the torso, neck, and peripheral arteries, each with unique diagnostic and management challenges. CTA remains the cornerstone for detecting these injuries, while endovascular and hybrid approaches are increasingly preferred for peripheral and thoracic aortic injuries, due to better limb salvage and reduced complications. Open repair, however, remains vital for complete transections and complex trauma. Future studies should focus on refining management strategies and evaluating long-term outcomes for different vascular injury patterns.
